# Prevalence of and factors associated with late diagnosis of HIV in Malawi, Zambia, and Zimbabwe: Results from population-based nationally representative surveys

**DOI:** 10.1371/journal.pgph.0000080

**Published:** 2022-02-22

**Authors:** Andreas D. Haas, Elizabeth Radin, Sehin Birhanu, Andrea J. Low, Suzue Saito, Karampreet Sachathep, Shirish Balachandra, Julius Manjengwa, Yen T. Duong, Sasi Jonnalagadda, Danielle Payne, George Bello, Avi J. Hakim, Theo Smart, Nahima Ahmed, Juliana Cuervo-Rojas, Andrew Auld, Hetal Patel, Bharat Parekh, Daniel B. Williams, Danielle T. Barradas, Owen Mugurungi, Lloyd B. Mulenga, Andrew C. Voetsch, Jessica E. Justman

**Affiliations:** 1 ICAP, Columbia University, New York, New York, United States of America; 2 Institute of Social and Preventive Medicine, University of Bern, Bern, Switzerland; 3 Division of Global HIV and TB, Centers for Disease Control and Prevention, Atlanta, Georgia, United States of America; 4 Centers for Disease Control and Prevention, Harare, Zimbabwe; 5 Centers for Disease Control and Prevention, Lilongwe, Malawi; 6 Government of Malawi, Ministry of Health, Lilongwe, Malawi; 7 Faculty of Medicine, Pontificia Universidad Javeriana, Bogotá, Colombia; 8 Centers for Disease Control and Prevention, Lusaka, Zambia; 9 Government of Zimbabwe, Ministry of Health and Child Care, Harare, Zimbabwe; 10 Government of Zambia, Ministry of Health, Lusaka, Zambia; Makerere University, UGANDA

## Abstract

**Introduction:**

Late diagnosis of HIV (LD) increases the risk of morbidity, mortality, and HIV transmission. We used nationally representative data from population-based HIV impact assessment (PHIA) surveys in Malawi, Zambia, and Zimbabwe (2015–2016) to characterize adults at risk of LD and to examine associations between LD and presumed HIV transmission to cohabiting sexual partners.

**Methods:**

We estimated the prevalence of LD, defined as CD4 count <350 cells/μL, among adults newly diagnosed with HIV during the surveys and odds ratios for associated factors. We linked newly diagnosed adults (index cases) to their household sexual partners and calculated adjusted odds ratios for associations between LD of the index case, viral load of the index case, and duration of HIV exposure in the relationship, and the HIV status of the household sexual partner.

**Results:**

Of 1,804 adults who were newly diagnosed with HIV in the surveys, 49% (882) were diagnosed late. LD was associated with male sex, older age, and almost five times the odds of having an HIV-positive household sexual partner (adjusted odds ratio [aOR], 4.65 [95% confidence interval: 2.56–8.45]). Longer duration of HIV exposure in a relationship and higher viral load of the index case were both independently associated with higher odds of having HIV-positive household sexual partners. Individuals with HIV exposure of more than 5 years had more than three times (aOR 3.42 [95% CI: 1.63–7.18]) higher odds of being HIV positive than those with less than 2 years HIV exposure. The odds of being HIV positive were increased in individuals who were in a relationship with an index case with a viral load of 400–3499 copies/mL (aOR 4.06 [95% CI 0.45–36.46]), 3,500–9,999 copies/mL (aOR 11.32 [95% CI: 4.08–31.39]), 10,000–49,999 copies/mL (aOR 17.07 [95% CI: 9.18–31.72]), and ≥50,000 copies/mL (aOR 28.41 [95% CI: 12.18–66.28]) compared to individuals who were in a relationship with an index case with a viral load of <400 copies/mL.

**Conclusions:**

LD remains a challenge in Southern Africa and is strongly associated with presumed HIV transmission to household sexual partners. Our study underscores the need for earlier HIV diagnosis, particularly among men and older adults, and the importance of index testing.

## Introduction

Late diagnosis of HIV (LD) is common in sub-Saharan Africa. Two systematic reviews from 2013 and 2015 showed that about 70% of HIV-positive people who were diagnosed in health facilities and about 25%–40% of those diagnosed in their communities were diagnosed late (i.e., with CD4 count <350 cells/μL) [1, 2].

LD leads to late initiation of antiretroviral therapy (ART) and consequently increases the risk of morbidity, mortality, and HIV transmission [3, 4]. Two major international randomized clinical trials showed that early ART initiation reduced severe illness or death among HIV-positive adults by 57% and HIV transmission to serodiscordant partners by 93% compared with late ART initiation [3, 4].

Tailored and differentiated HIV testing approaches, including active index case testing and self-testing, are promising approaches to diagnose people living with HIV who are unaware of their status [5–9]. Active index testing is increasingly used to reach undiagnosed partners, especially male partners, with testing and treatment services and has a low cost per HIV-positive result [5, 10]. Other World Health Organization (WHO) recommended screening or testing approaches to reach undiagnosed individuals include primary or secondary distribution of self-tests or targeted mobile testing [7–11].

Nationally representative estimates of the prevalence of LD and associated factors can inform HIV testing strategies to improve individual and population health outcomes. However, current estimates for LD prevalence are from non-representative studies conducted in selected communities or health facilities; furthermore, there are no data on associations between LD and HIV transmission [1, 2].

We analyzed data from population-based HIV impact assessments (PHIAs) to calculate nationally representative estimates of the prevalence and burden of LD in Malawi, Zambia, and Zimbabwe. We characterized populations at risk of LD to guide targeting of HIV-testing services and studied associations between LD and HIV status of HIV-exposed household sexual partners.

## Methods

### Study design

PHIA surveys are nationally-representative, cross-sectional household surveys conducted in 13 African countries and Haiti [12, 13]. Survey participants completed questionnaires and were offered home-based HIV counselling and testing. People diagnosed with HIV received confirmatory HIV testing, testing for recent HIV infection using an HIV-1 limiting antigen (LAg)-avidity assay, and additional biomarker testing, including CD4 cell count testing, HIV viral load testing and testing for the presence of selected antiretroviral drugs (ARVs) using a qualitative high-performance liquid chromatography/tandem mass spectrometry assay [14–16]. Additional information on laboratory methods used to detect the presence on ARVs are provided in the appendix (S1 Text). Samples for all blood tests were collected on the same day. Additional information regarding the surveys has been published elsewhere [17–19]. Questionnaires are available online, under https://phia-data.icap.columbia.edu/files.

### Outcomes

LD was defined as being newly diagnosed with HIV by the survey team at a CD4 count <350 cells/μL. HIV-positive status of exposed partners was based on HIV testing conducted during the survey.

### Procedures and participants

**Fig 1:**
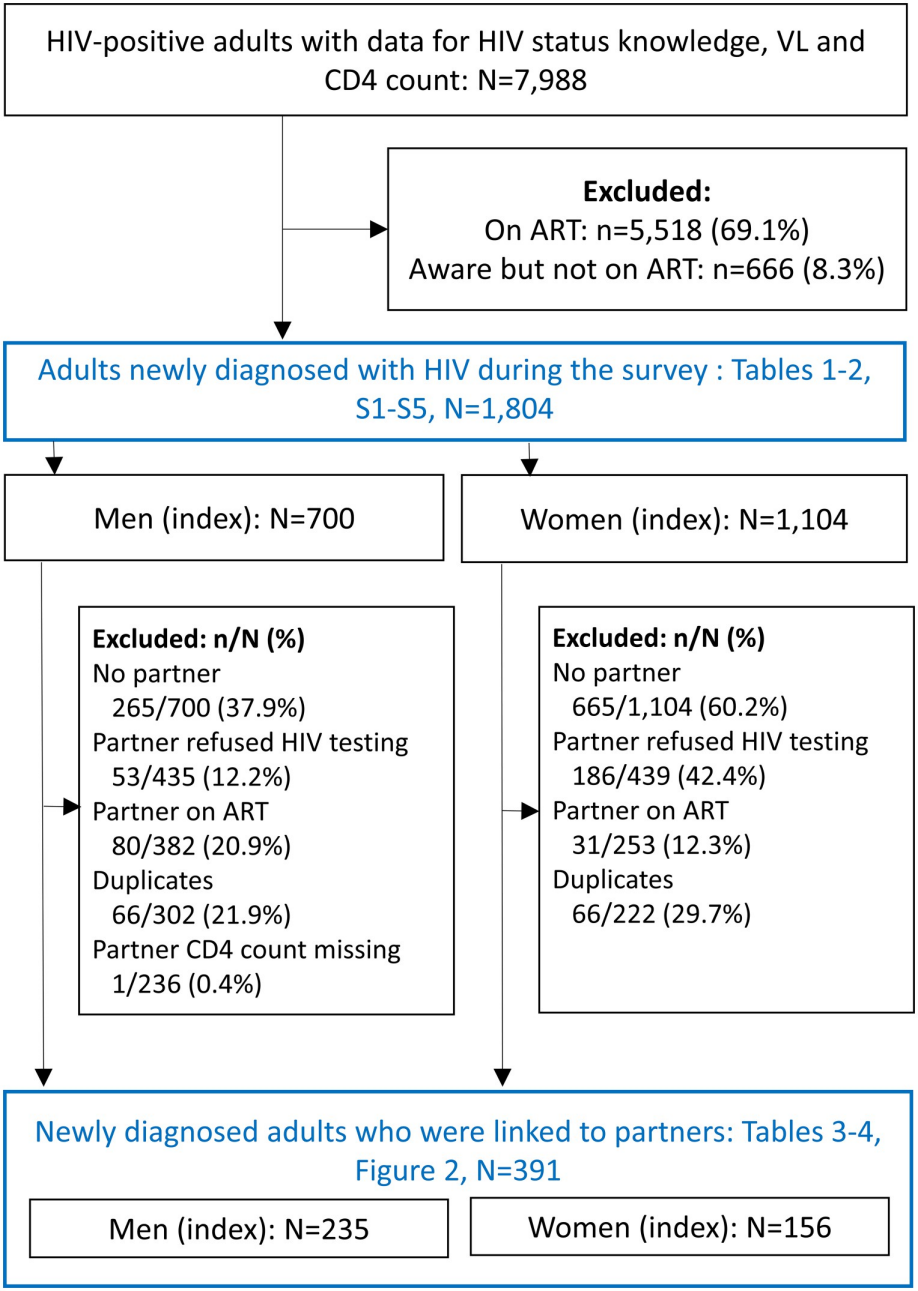
Adults diagnosed with HIV during PHIA surveys in Malawi, Zambia, and Zimbabwe (2015–2016) and their sexual partners included in analysis of late diagnosis of HIV. Flowchart showing unweighted percentages of survey participants excluded from analysis by reason for ineligibility. Abbreviations: PHIA, population-based HIV impact assessment; N, denominator; n, numerator; VL, viral load; ART, antiretroviral therapy.

Adults who were newly diagnosed with HIV during the PHIA survey (i.e., who tested HIV positive during the survey, reported a negative or unknown HIV status, and had no detectable ARVs in their blood) were eligible for analysis of the prevalence of LD and associated factors. Participants with missing CD4 count or viral load test result were excluded (Fig 1). Adults were defined according to countries’ definitions (15–64 years in Malawi and Zimbabwe, and 15–59 years in Zambia).

We linked adults who were newly diagnosed with HIV during the survey (index case) to their household sexual partners to assess associations between LD among index cases and the HIV status of their partners (Fig 1). Couples in which the partner refused HIV testing were excluded. We used a CD4 depletion model to estimate time since seroconversion, and inferred the direction of HIV transmission among couples based on the time since seroconversion of both partners [20, 21]. We excluded couples in which the partners had initiated ART because time since seroconversion can only be estimated for ART-naïve people. Couples in which both partners were newly diagnosed during the PHIA survey appeared twice in the linked dataset (i.e., the same person appeared as index case and as linked partner). We deduplicated the dataset by removing couples in which the estimated date of seroconversion of the linked partner preceded the estimated date of seroconversion of the index case. We analyzed the deduplicated dataset to examine associations between LD of index cases, viral load of index cases, and the duration of HIV exposure in a relationship, and the HIV status of their partners. Viral load was categorized as <400 copies/mL, 400–3,499 copies/mL, 3,500–9,999 copies/mL, 10,000–49,999 copies/mL, and ≥50,000 copies/mL. Duration of HIV exposure in a relationship was defined as the shortest of two periods: the estimated time since seroconversion of the index case, and the duration of the relationship, and categorized into <2 years, 2–5 years, and >5 years.

### Statistical analysis

We used summary statistics to describe characteristics of adults newly diagnosed with HIV and their household sexual partners. We calculated unadjusted (OR) and adjusted odds ratios (aOR) and 95% confidence intervals (CI) for factors associated with LD using logistic regression. We used purposeful selection of covariates to build regression models for factors associated with LD [22]. We considered key sociodemographic characteristics, indicators for HIV testing, sexual risk behavior, HIV knowledge, discriminatory attitude toward people living with HIV, and perceived HIV stigma in unadjusted analysis. Variables associated with LD at a p-value of <0.2 in unadjusted analysis were considered in multivariable analysis. Variables not associated at a p-value of <0.05 in multivariable analysis were eliminated from the final model [22]. All multivariable models adjusted for country.

We calculated the average time since seroconversion for all treatment-naïve HIV-positive adults based on estimates from a linear mixed model of the natural history of CD4 depletion among sub-Saharan African adults by age and sex [20]. Per the model presented by Song et al., time since seroconversion was calculated as the difference of the square root of the CD4 count observed at the time of survey participation and the square root of the calculated CD4 at seroconversion divided by the square root of the annual CD4 depletion [21]. As in Song et al., implementing this model results in a negative time since seroconversion for individuals with observed CD4 count above the modeled CD4 at seroconversion. We reset negative time since seroconversion with plausible values: 65 days (the midpoint of the mean duration of recent infection [MDRI] for the LAg avidity assay, i.e., 130 days) if a participant tested positive for recent HIV infection and 131 days (i.e., 1 day after MDRI) if a participant tested negative for recent HIV infection.

We fitted logistic regression models to assess factors associated with the HIV status of household sexual partners of HIV-positive index cases. The CD4 count and viral load of the index case and the duration of HIV exposure were not linearly related to the log odds of HIV status among exposed partners. We used two analytic approaches to address the violation of the non-linearity assumption of logistic regression. First, we calculated unadjusted and aORs for categorical predictors of these factors. Second, we fitted a non-linear model with restricted cubic splines with knots at the 10th, 50th and 90th percentiles for continuous predictors of CD4 count of index cases and calculated aORs for HIV-positive status among exposed partners.

All analyses were weighted for sampling selection probabilities, noncoverage, and nonresponse and accounted for stratification and clustering in the sample design. Variance was estimated using the Taylor series linearization method. We report weighted percentages, and unweighted numbers of participants if not otherwise stated. Medians and interquartile ranges (IQRs) are weighted. Stata (Version 15, Stata Corporation, College Station, Texas, United States) was used for statistical analysis.

### Ethical considerations

The National Health Science Research Committee Malawi, the Tropical Diseases Research Centre Ethics Review Committee, Zambia, the Medical Research Council of Zimbabwe, and the Institutional Review Boards at the Centers for Disease Control and Prevention (CDC; Atlanta, Georgia, United States) and Columbia University Medical Center (New York, New York, United States) approved the PHIA surveys. All participants provided written informed consent. Additional information regarding consent procedures has been published elsewhere [17–19].

## Results

Of 7,988 HIV-positive adults included in the surveys, 25% (1,804) were newly diagnosed with HIV during the survey, 8% (666) were diagnosed before the survey but had not initiated ART, and 67% (5,518) were receiving ART. The 1,804 adults newly diagnosed with HIV were eligible for our analysis of prevalence of LD and associated factors (Fig 1).

Characteristics of adults newly diagnosed with HIV are shown in Table 1. Most were women (55%), lived in rural areas (58%), were married or cohabitating (60%), and had completed at least secondary education (52%). Median age was 32 years (IQR, 26–42 years) (Table 1). Prevalence of sexual risk behavior, comprehensive HIV knowledge, and HIV-related stigma among HIV-positive adults unaware of their status are shown in S1 Table. Median time since seroconversion was estimated to be 3.4 years (IQR, 0.4–4.2 years) (S2 Table). Median CD4 count among HIV-positive people unaware of their status was 356 cells/μL (IQR, 231–508 cells/μL).

**Table 1:** Sociodemographic characteristics of adults diagnosed with HIV during the survey in Malawi, Zambia, and Zimbabwe (2015–2016)^a^.

	CD4<350 cells/μL Late diagnosis, n (%)^a^	CD4≥350 cells/μL, n (%)^a^	Total, n (%)^a^
	N = 882	N = 922	N = 1,804
Country	882 (100.0)	922 (100.0)	1,804 (100.0)
Malawi	223 (26.1)	249 (27.7)	472 (26.9)
Zambia	301 (34.0)	347 (38.8)	648 (36.5)
Zimbabwe	358 (39.9)	326 (33.5)	684 (36.6)
Residence	882 (100.0)	922 (100.0)	1,804 (100.0)
Urban	394 (42.7)	418 (41.2)	812 (41.9)
Rural	488 (57.3)	504 (58.8)	992 (58.1)
Gender	882 (100.0)	922 (100.0)	1,804 (100.0)
Male	393 (50.8)	307 (39.9)	700 (45.2)
Female	489 (49.2)	615 (60.1)	1,104 (54.8)
Age, years	882 (100.0)	922 (100.0)	1,804 (100.0)
15–24	127 (15.4)	244 (26.4)	371 (21.0)
25–34	299 (35.3)	320 (36.0)	619 (35.7)
35–44	250 (27.9)	208 (22.2)	458 (25.0)
45–65	206 (21.4)	150 (15.5)	356 (18.4)
Median (IQR)^b^	34 (28–43)	30 (24–40)	32 (26–42)
Marital status	880 (100.0)	922 (100.0)	1,802 (100.0)
Never married	120 (14.0)	179 (20.4)	299 (17.3)
Married or living together	520 (61.0)	529 (58.2)	1,049 (59.6)
Divorced, separated	143 (15.8)	149 (15.7)	292 (15.7)
Widowed	97 (9.2)	65 (5.6)	162 (7.4)
Education	882 (100.0)	920 (100.0)	1,802 (100.0)
No or primary	427 (48.4)	440 (48.3)	867 (48.4)
Secondary or higher	455 (51.6)	480 (51.7)	935 (51.6)
Wealth quintile^c^	882 (100.0)	920 (100.0)	1,802 (100.0)
Lowest	143 (16.4)	145 (15.9)	288 (16.1)
Second	141 (16.4)	139 (16.2)	280 (16.3)
Middle	160 (18.6)	179 (20.1)	339 (19.4)
Fourth	191 (23.3)	215 (25.6)	406 (24.4)
Highest	247 (25.3)	242 (22.2)	489 (23.7)

^a^ Data are unweighted numbers of participants and (weighted percentages) unless otherwise stated.

^b^ Medians and IQRs are weighted.

^c^ We used an asset-based principal component wealth index to calculate a wealth index score and defined household wealth according to the wealth index score quintiles.

Abbreviations: IQR, interquartile range.

The prevalence and weighted number of persons diagnosed late during the survey by age, sex, and country are shown in Table 2. Half of the people newly diagnosed during the survey (49%) met criteria for LD (men, 55%; women, 44%). This corresponds to a weighted number of 355,335 undiagnosed adults (men, 180,346; women, 174,989) meeting LD criteria in the total population of the three countries (Table 2).

**Table 2:** Prevalence and weighted number persons with late diagnosis of HIV during the survey in Malawi, Zambia, and Zimbabwe (2015–2016).

	CD4<350 cells/μL Late diagnosis	CD4 ≥350 cells/μL	Total
	N = 882^a^ Weighted N (weighted %)^b^	N = 922^a^ Weighted N (weighted %)^b^	N = 1,804^a^ Weighted N (weighted %)^b^
Country	355,335 (48.8)	373,213 (51.2)	728,548 (100.0)
Malawi	92,757 (47.3)	103,272 (52.7)	196,029 (100.0)
Zambia	120,917 (45.5)	144,789 (54.5)	265,706 (100.0)
Zimbabwe	141,661 (53.1)	125,151 (46.9)	266,813 (100.0)
Sex	355,335 (48.8)	373,213 (51.2)	728,548 (100.0)
Male	180,346 (54.8)	148,978 (45.2)	329,324 (100.0)
Female	174,989 (43.8)	224,234 (56.2)	399,224 (100.0)
Age, years	355,335 (48.8)	373,213 (51.2)	728,548 (100.0)
15–24	54,733 (35.7)	98,432 (64.3)	153,165 (100.0)
25–34	125,483 (48.3)	134,326 (51.7)	259,809 (100.0)
35–44	99,076 (54.5)	82,706 (45.5)	181,782 (100.0)
45–65	76,044 (56.8)	57,748 (43.2)	133,792 (100.0)

^a^ N in table header are unweighted numbers of participants.

^b^ Data are weighted numbers of participants and (weighted percentages).

Country, sex, age, and marital status were associated with LD at a significance level α<0.2 in unadjusted analysis and considered in multivariable analysis (S3 and S4 Tables). In multivariable analysis, LD was associated with male sex (aOR, 1.40 [95% CI: 1.10–1.78]) and older age: aORs for LD were 1.59 (95% CI: 1.10–2.29) for people aged 25–34 years, 1.96 (95% CI: 1.32–2.90) for people aged 35–44 years, and 2.19 (95% CI: 1.48–3.26) for people aged 45–65 years compared to people aged 15–25 years (Table 3).

**Table 3:** Multivariable analysis of factors associated with late HIV diagnosis in Malawi, Zambia, and Zimbabwe (2015–2016)^a^.

	aOR (95% CI)	p-value^a^
Country		0.152
Malawi	1.00	
Zambia	0.99 (0.70–1.40)	
Zimbabwe	1.27 (0.90–1.79)	
Age in years		0.001
15–24	1.00	
25–34	1.58 (1.09–2.30)	
35–44	1.95 (1.32–2.89)	
45–65	2.18 (1.47–3.23)	
Sex		0.009
Male	1.38 (1.08–1.76)	
Female	1.00	

^a^ P-values of categorical variables are for joint test for significance. Analysis adjusted for age, sex, and country.

Abbreviations: aOR, adjusted odds ratio; CI, confidence interval.

Of the 391 couples eligible for our analysis of factors associated with HIV status of household sexual partners, 261 were serodiscordant, and 130 were HIV-positive seroconcordant couples. Having no partner, partners’ refusal of HIV testing, or partners’ use of ART were frequent reasons for ineligibility. More male than female partners refused HIV testing, and more female than male partners received ART (Fig 1).

Characteristics of adults who were newly diagnosed with HIV during the PHIA survey (index case) and their household sexual partners are shown in Table 4. Most index cases were men (67%), and median age was 35 years (IQR, 30–42). Median viral load was slightly higher among index cases with CD4<350 cells/μL (4.7 log_10_ copies/mL; IQR, 4.4–5.2) than among those with CD4≥350 cells/μL (4.2 log_10_ copies/mL; IQR, 3.4–4.8). The median duration of HIV-exposure was 6.0 years (IQR, 4.3–8.3 years) among partners of individuals who were newly diagnosed at CD4<350 cells/μL, and 0.9 years (IQR, 0.4–2.2 years) among partners of those newly diagnosed at CD4≥350 cells/μL. HIV prevalence was 47% among exposed partners of individuals who were diagnosed at CD4<350 cells/μL, and 18% among those in a relationship with individuals who were diagnosed at CD4**≥**350 cells/μL. Among HIV-positive seroconcordant couples, the CD4 cell count of the index case was on average 194 cells/μL lower than the CD4 count of partner (IQR, 99–350 cells/μL lower).

**Table 4:** Characteristics of partnered adults newly diagnosed with HIV during the PHIA surveys and who had a partner who agreed to HIV testing and was not on ART^a^.

	CD4<350 cells/μL Late diagnosis	CD4 ≥350 cells/μL	Total
	N = 226	N = 165	N = 391
**Characteristics of adults diagnosed with HIV during the survey (index case)**			
Country	226 (100.0)	165 (100.0)	391 (100.0)
Malawi	51 (23.3)	51 (32.7)	102 (27.4)
Zambia	77 (33.1)	53 (31.9)	130 (32.6)
Zimbabwe	98 (43.6)	61 (35.4)	159 (40.0)
Sex	226 (100.0)	165 (100.0)	391 (100.0)
Male	137 (66.9)	98 (66.9)	235 (66.9)
Female	89 (33.1)	67 (33.1)	156 (33.1)
Age, years	226 (100.0)	165 (100.0)	391 (100.0)
15–24	21 (8.7)	17 (8.0)	38 (8.4)
25–34	84 (41.1)	62 (41.9)	146 (41.5)
35–44	67 (27.9)	62 (35.5)	129 (31.2)
45–65	54 (22.3)	24 (14.6)	78 (18.9)
Median (IQR)^b^	35 (29–43)	35 (30–40)	35 (30–42)
CD4, cells/μL	226 (100.0)	165 (100.0)	391 (100.0)
<100	31 (13.1)	0 (0.0)	31 (7.4)
100–199	74 (32.1)	0 (0.0)	74 (18.1)
200–349	121 (54.8)	0 (0.0)	121 (31.0)
350–499	0 (0.0)	89 (54.8)	89 (23.8)
≥500	0 (0.0)	76 (45.2)	76 (19.7)
Median (IQR)^b^	216 (143–278)	485 (412–611)	314 (199–452)
Viral load category, copies/mL	224 (100.0)	163 (100.0)	387 (100.0)
<400	6 (3.2)	20 (13.6)	26 (7.7)
400–3,499	9 (3.8)	22 (13.8)	31 (8.2)
3,500–9,999	13 (5.8)	23 (13.9)	36 (9.3)
10,000–49,999	83 (34.0)	56 (32.2)	139 (33.2)
≥50,000	113 (53.2)	42 (26.5)	155 (41.6)
Viral load in log_10_ copies/mL, median (IQR)^b^	4.7 (4.4–5.2)	4.2 (3.4–4.8)	4.6 (4.0–5.0)
**Characteristics of sexual partners**			
Sex	226 (100.0)	165 (100.0)	391 (100.0)
Male	89 (33.1)	67 (33.1)	156 (33.1)
Female	137 (66.9)	98 (66.9)	235 (66.9)
Age, years	226 (100.0)	165 (100.0)	391 (100.0)
15–24	43 (22.5)	23 (17.9)	66 (20.5)
25–34	87 (38.8)	73 (46.1)	160 (42.0)
35–44	57 (23.2)	52 (26.9)	109 (24.8)
45–65	39 (15.5)	17 (9.0)	56 (12.7)
Median (IQR)^b^	32 (26–41)	31 (26–38)	32 (26–39)
Duration of HIV exposure in relationship, years	226 (100.0)	165 (100.0)	391 (100.0)
<2	18 (6.7)	117 (72.4)	135 (35.3)
2–5	67 (32.1)	48 (27.6)	115 (30.1)
>5	141 (61.2)	0 (0.0)	141 (34.6)
Median (IQR)^b^	6.0 (4.3–8.3)	0.9 (0.4–2.2)	3.6 (1.1–6.5)
HIV status	226 (100.0)	165 (100.0)	391 (100.0)
Negative	125 (53.2)	136 (82.4)	261 (65.9)
Positive	101 (46.8)	29 (17.6)	130 (34.1)
**Characteristics of HIV-positive seroconcordant couples**	N = 101	N = 29	N = 130
CD4 count difference: partner-index in μL, median (IQR)	217 (109–428)	113 (55–255)	194 (99–350)
Time from seroconversion of index case to seroconversion of partner, years	101 (100.0)	29 (100.0)	130 (100.0)
<1	10 (10.4)	15 (47.9)	25 (18.8)
1–2	10 (8.9)	12 (45.8)	22 (17.2)
2–4	25 (25.3)	2 (6.3)	27 (21.1)
4–6	23 (20.4)	0 (0.0)	23 (15.8)
≥6	33 (35.0)	0 (0.0)	33 (27.1)
Median (IQR)^b^	4.4 (2.3–6.5)	1.0 (0.2–1.2)	3.3 (1.2–6.4)

^a^ Data are number of participants and (weighted percentages) if not stated otherwise.

^b^ Medians and IQRs are weighted. Abbreviations: IQR, interquartile range.

**Fig 2:**
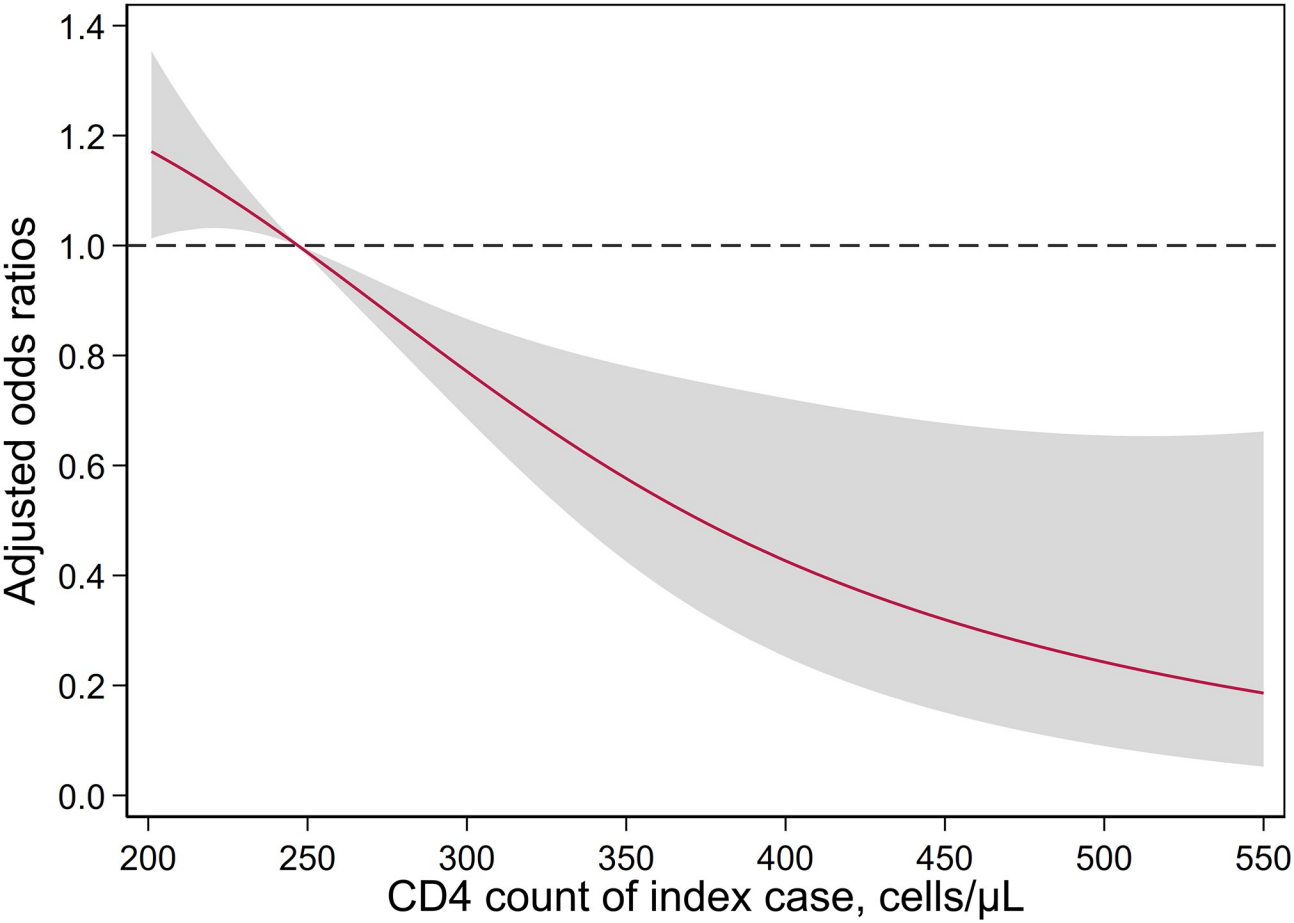
Adjusted odds ratios for HIV-positive status among exposed partners by CD4 count depletion of adults diagnosed with HIV during the survey (index case) in Malawi, Zambia, and Zimbabwe (2015–2016). Exposed partners of index cases with CD4 count of 250 cells/μL are the reference group. Odds ratios are adjusted for sex and country. Shaded area shows 95% confidence interval.

A CD4 count of less than 350 cells/μL among index cases, the criterion for LD, was associated with more than four times the odds for HIV-positive status among exposed household sexual partners (OR, 4.51 [95% CI: 2.41–8.44]). After adjusting for country and sex, the association between LD and HIV-positive status among exposed partners was similar (aOR, 4.59 [95% CI: 2.50–8.46]). Partners of index cases who were diagnosed with a CD4 count of 500 cells/μL and 350 cells/μL had 76% (aOR, 0.24 [95% CI: 0.09–0.65]) and 43% (aOR, 0.57 [95% CI: 0.42–0.78]) lower odds of being HIV-positive than those who were in a relationship with persons diagnosed with a CD4 count of 250 cells/μL, respectively (Fig 2).

Partners with HIV exposure of more than 5 years had almost five times the odds of being HIV-positive (OR, 4.81 [95% CI: 2.36–9.79]) than partners with less than 2 years of HIV exposure. The aOR for HIV-positive status comparing >5 and <2 years HIV exposure decreased to 3.42 (95% CI: 1.63–7.18) after we adjusted for viral load of the index case, sex, and country. Higher viral load in index cases was strongly associated with greater odds of a positive HIV status among exposed partners in both unadjusted and adjusted analysis. The odds of being HIV positive were significantly increased in individuals who were in a relationship with an index case with a viral load of 3,500–9,999 copies/mL (aOR 11.32 [95% CI: 4.08–31.39]), 10,000–49,999 copies/mL (aOR 17.07 [95% CI: 9.18–31.72]), and ≥50,000 copies/mL (aOR 28.41 [95% CI: 12.18–66.28]) compared to individuals who were in a relationship with an index case with a viral load of <400 copies/mL. Exposed women had lower adjusted odds for HIV-positive status (aOR, 0.52 [95% CI: 0.30–0.91]) than exposed men after adjusting for viral load, duration of exposure, and country (Table 5).

**Table 5:** Factors associated with HIV-positive status of exposed partners of adults newly diagnosed with HIV during the survey in Malawi, Zambia, and Zimbabwe (2015–2016).

	Unadjusted analysis		Multivariable analysis of late diagnosis		Multivariable analysis of duration of HIV exposure and viral load	
	OR (95% CI)	p-value^a^	aOR (95% CI)	p-value^a^	aOR (95% CI)	p-value^a^
**Characteristics of adults newly diagnosed with HIV during the survey (index case)**						
CD4 cells/μL		<0.001		<0.001		
≥350	1.00		1.00			
<350 (Late diagnosis)	4.51 (2.41–8.44)		4.59 (2.50–8.46)			
Viral load of index case, copies/mL		<0.001				<0.001
<400	1.00				1.00	
400–3499	4.06 (0.45–36.46)				4.37 (0.44–43.38)	
3,500–9,999	10.96 (4.38–27.44)				11.32 (4.08–31.39)	
10,000–49,999	17.88 (9.69–33.01)				17.07 (9.18–31.72)	
≥50,000	29.17 (16.54–51.46)				28.41 (12.18–66.28)	
**Duration of HIV exposure in relationship, years**		<0.001				
<2	1.00				1.00	0.006
2–5	2.71 (0.90–8.20)				2.08 (0.71–6.08)	
>5	4.81 (2.36–9.79)				3.42 (1.63–7.18)	
**Country**		0.817		0.875		0.929
Malawi	1.00		1.00		1.00	
Zambia	1.18 (0.52–2.67)		1.09 (0.56–2.12)		0.87 (0.38–2.01)	
Zimbabwe	1.31 (0.56–3.09)		1.22 (0.56–2.65)		0.97 (0.40–2.35)	
**Characteristics of partners**						
Sex				0.049		0.024
Male	1.00	0.126	1.00		1.00	
Female	0.66 (0.39–1.11)		0.59 (0.35–1.00)		0.52 (0.30–0.91)	
Age, years		0.672				
15–24	1.00					
25–34	1.29 (0.53–3.14)					
35–44	0.85 (0.37–1.97)					
45–65	0.88 (0.28–2.76)					

^a^ P-values of categorical variables are for joint test for significance. Multivariable analysis adjusted for all variables shown in the model.

Abbreviations: OR, odds ratio; aOR, adjusted odds ratio; CI, confidence interval.

## Discussion

This is the first multicountry, nationally representative population-based study of the prevalence of LD of HIV and associated factors. Of the one-quarter who were unaware of their HIV-positive status among all HIV-positive participants in the surveys, half had LD. Thus, LD is common in high prevalence settings and affects over 12% of all HIV-positive adults. Additionally, half of the household sexual partners of LD individuals were HIV-positive, with the odds of HIV-positive status inversely related to the CD4 count of the index case. LD was associated with more than four times the odds of HIV-positive status among HIV-exposed household sexual partners. Partners of undiagnosed people with a CD4 count of 500 cells/μL had 76% lower odds of being HIV positive than partners of undiagnosed people with a CD4 count of 250 cells/μL. For exposed partners, the odds of being HIV-positive were associated with both the duration of HIV-exposure and the viral load of the index partner.

HIV diagnosis is the crucial first step of the 95-95-95 HIV care cascade [23]. UNAIDS launched the 95-95-95 strategy to put the world on the trajectory to end the AIDS epidemic by 2030, with the aim that by 2025, 95% of people living with HIV are diagnosed, 95% of those diagnosed are receiving ART, and 95% of those receiving ART achieve virological suppression. The high prevalence of people living with HIV diagnosed late during the PHIA survey points to the urgent need to close gaps in HIV testing coverage to reach the first “95” target. The success of the 95-95-95 strategy crucially depends on closing gaps in HIV testing coverage.

The high prevalence of LD suggests that traditional facility-based HIV testing strategies have failed to reach a sizeable group of people in need of testing. This suggests a need to prioritize new and innovative approaches to diagnosis. The implementation of differentiated community-based HIV testing strategies is recommended to reach groups missed by facility-based HIV-testing services [24]. Differentiated HIV testing strategies adjust the test modalities (i.e., the where, by whom, and when HIV testing is offered) to reflect the preferences, expectations, and needs of various groups of people living with HIV [25, 26]. For example, to reach men in South Africa, HIV testing services were offered by male community health workers outside of working hours at locations where men frequently gather [27]. Another promising strategy that can be integrated in differentiated HIV testing approaches is index testing. Index testing offers HIV testing to sexual partners and children of persons diagnosed with HIV [5, 6, 28, 29]. About one-third of the men newly diagnosed during the PHIA survey were in a relationship with an HIV-positive woman who already knew her status [30]. Index testing programs could have efficiently reached these undiagnosed men through their partners [29]. Increasingly used in many African countries, the strategy has been found to be an efficient approach to identify undiagnosed people living with HIV [5, 28]. For example, index testing has been piloted successfully in three provinces in Zimbabwe: Between 2016 and 2018, almost 16,000 people living with HIV were diagnosed through index testing; the HIV positivity rate for index texting was 29% compare to 4% for provider-initiated facility-based HIV testing and counselling [6]. Index testing has also been piloted successfully in Zambia, where an HIV positive rate of 40% in men and 49% in women was achieved [31]. Finally, HIV self-testing is another promising strategy to increase the HIV testing coverage in sub-Saharan Africa. A systematic review of 14 randomized controlled trials showed that HIV self-testing doubled the uptake of HIV testing compared to provider-initiated facility-based HIV testing [8]. Differentiated models of care for persons diagnosed with advanced HIV disease can also be leveraged for reducing HIV-related morbidity and mortality. The World Health Organization (WHO) recommends screening, treatment and prophylaxis for major opportunistic infections, rapid ART initiation, and enhanced ART adherence support for adults and adolescents, and children ≥5 years old, presenting with a CD4 cell count <200 cells /μL or a WHO clinical stage 3 or 4 event [32]. Recommended diagnostic services and prophylactic therapies for persons with advanced HIV disease include symptomatic screening for cryptococcal meningitis, mycobacterial lipoarabinomannan testing, cryptococcal antigen testing, fluconazole prophylaxis, cotrimoxazole prophylaxis, and tuberculosis prophylactic treatment [32]. These services can be integrated into differentiated service delivery models [33, 34].

Our findings suggest that intensified efforts to diagnose and treat HIV-positive people earlier could help prevent HIV transmission in serodiscordant partnerships and reduce morbidity and mortality among HIV-positive people. We found a strong association between late HIV diagnoses of index cases and HIV-positive status among exposed partners. This association is likely caused by two underlying mechanisms: high infectiousness of people with low CD4 owing to high viral loads in late-stage infection [35, 36] and an increasing cumulative risk of HIV transmission among partners of people with low CD4 owing to their longer duration of infection and consequently longer HIV exposure for HIV-negative partners. This finding is in agreement with evidence showing that the viral load of an HIV-positive partner is a crucial determinant for the risk of HIV transmission between serodiscordant sexual partners [37] and underscores the importance of diagnosing and treating HIV early to decrease the duration of HIV exposure in serodiscordant relationships. While the association between high viral load and HIV transmission is well established in the literature [37], there is little emphasis on the accumulation of risk over a long duration of HIV exposure among serodiscordant couples. Our findings show that the duration of HIV exposure in a relationship is independently associated with HIV-positive status among serodiscordant couples regardless of the viral load of the HIV-positive person. Our real world data are in agreement with the HPTN 052 HIV prevention trial showing that early ART initiation at a median CD4 cell count of 442 cells/μL compared to late ART initiation at a CD4 cell count of less than 250 cells/μL reduced HIV transmission (genetically linked and unlinked) among serodiscordant couples by 89% [4].

One important strength of this analysis is the use of nationally- representative data from three southern African countries heavily affected by HIV. A further strength of our study is that our definition of awareness of HIV status incorporated both self-report and the results of ARV detection assays. A final strength is that we linked HIV-positive people and their household sexual partners. We identified the index case as the partner with the longer estimated time since seroconversion, which enabled us to study associations between LD and HIV-positive status among HIV-exposed sexual partners. A novel feature of this analysis was the use of an established CD4 depletion model to estimate the average time since seroconversion based on CD4 data from sub-Saharan Africa by age and sex [20, 21, 38, 39].

Our results should be considered in light of several limitations. The CD4 depletion model does not account for individual variability in CD4 cell counts nor variability in CD4 cell count depletion owing to other factors including alcohol use, pregnancy, other infections, or other chronic conditions [38, 40–44]. Inaccurate estimation of the time since seroconversion could lead to misclassification of the direction of transmission and misclassification bias. A further limitation of our analysis of factors associated with HIV-positive status is that we may have introduced selection bias by excluding newly diagnosed individuals who were linked to partners receiving ART. We excluded these couples because we could not estimate the time since seroconversion for patients receiving ART and hence could not identify the index case within these couples. Our data showed an unexpected association between male sex and the odds of HIV-positive status. The exclusion of more HIV-positive female partners receiving ART (21%) than HIV-positive male partners receiving ART (12%) due to higher ART coverage among women likely explains this unexpected finding. A further limitation of our study is that we could not determine whether HIV transmission had occurred within the couple or from an additional sexual partner not living in the household because we lacked molecular sequence data to genetically link transmission pairs. Given the inability to distinguish the origin of the infection of the partner (our index case or external partners) our estimate of the association between LD and infection of the partner may be biased.

## Conclusions

Timely diagnosis remains a challenge in Southern Africa. Our findings suggest that scaling up tailored and targeted HIV-testing programs for earlier diagnosis of HIV and earlier ART initiation, particularly among men and older adults, could improve individual outcomes and prevent HIV transmission.

## Supporting information

S1 Text Laboratory methods for assessing the presence of antiretroviral drugs. (DOCX)

S1 Table Prevalence of sexual risk behavior, comprehensive HIV knowledge, and perceived HIV-related stigma among adults newly diagnosed with HIV during the survey in Malawi, Zambia, and Zimbabwe (2015–2016). (DOCX)

S2 Table Estimated median (interquartile range) time (years) since seroconversion among men and women newly diagnosed with HIV during the survey in Malawi, Zambia, and Zimbabwe (2015–2016). (DOCX)

S3 Table Unadjusted analysis of sociodemographic factors associated with late diagnosis of HIV in Malawi, Zambia, and Zimbabwe (2015–2016). (DOCX)

S4 Table Unadjusted analysis of associations between sexual risk behavior, HIV knowledge, and HIV-related stigma and late diagnosis of HIV in Malawi, Zambia, and Zimbabwe (2015–2016). (DOCX)
