# Supplementary material for: Prevalence of and factors associated with late diagnosis of HIV in Malawi, Zambia, and Zimbabwe: Results from population-based nationally representative surveys
Source: PLOS Glob Public Health. 2022 Feb 22;2(2):e0000080. doi: 10.1371/journal.pgph.0000080 (PMC10021857; doi:10.1371/journal.pgph.0000080)
Supplement: S1 Text — (DOCX) [file pgph.0000080.s001.docx]

**S1 Text: Laboratory methods for processing of HIV-positive specimens**

All HIV-positive results were laboratory-confirmed using Bio-Rad Geenius HIV1/2 (Bio-Rad, Hercules, CA). People who tested HIV-seropositive received PIMA point-of-care CD4 testing (Alere, Waltham, MA) with immediate return of results and submitted plasma specimens for HIV-1 RNA VL testing using the Abbott m2000 System (Abbott Molecular Inc., Chicago, IL) or the Roche COBAS AmpliPrep/COBAS TaqMan platform (Roche Molecular Diagnostics, Pleasanton, CA). The same platforms were used to test dried blood spots (DBS) for VL, in addition to the NucliSENSTM EasyQ HIV-1 v2.0 assay on the bioMérieux (Marcy-l’Étoile, France) for Zimbabwe.

A qualitative high-performance liquid chromatography/tandem mass spectrometry assay was performed to detect antiretroviral drug (ARV) in dried blood spots at the Division of Clinical Pharmacology, University of Cape Town. The samples were processed with a protein precipitation extraction method. Deuterated internal standards were used for each analyte. The extraction procedure was followed by liquid chromatographic separation using a Phenomenex Kinetex EVO C18 (Phenomenex Helvetia, Basel, Switzerland) (1.7 μm; 2.1 × 50 mm; 100 Å) analytical column. An AB Sciex API 4000 mass spectrometer (SCIEX, Framingham, MA, USA) at unit resolution in the multiple reaction monitoring mode was used to monitor the transition of the protonated precursor ions m/z 705.6, 316.0, 629.6, and 267.1 to the product ions m/z 168.2, 243.9, 447.3, and 226.0 for atazanavir, efavirenz, lopinavir, and nevirapine respectively. Electro Spray ionisation was used for ion production. The assay was validated over the range of 0.02–5.0 μg/mL, and 0.02 μg/mL was used as the cut-off concentration.
