# Supplementary material for: Prevalence of and factors associated with late diagnosis of HIV in Malawi, Zambia, and Zimbabwe: Results from population-based nationally representative surveys
Source: PLOS Glob Public Health. 2022 Feb 22;2(2):e0000080. doi: 10.1371/journal.pgph.0000080 (PMC10021857; doi:10.1371/journal.pgph.0000080)
Supplement: S1 Table — (DOCX) [file pgph.0000080.s002.docx]

**S1 Table: Prevalence of sexual risk behavior, comprehensive HIV knowledge, and perceived HIV-related stigma among adults newly diagnosed with HIV during the survey in Malawi, Zambia, and Zimbabwe (2015–2016)^a^**

|  | **Late diagnosis**  **CD4<350 cells/µL** | **CD4≥350 cells/µL** | **Total** |
| --- | --- | --- | --- |
|  | N=882 | N=922 | N=1,804 |
| Buying or selling sex in the 12 months before the survey, n (%) | 650 (100.0) | 677 (100.0) | 1,327 (100.0) |
| Yes | 35 (5.9) | 36 (5.2) | 71 (5.6) |
| No | 615 (94.1) | 641 (94.8) | 1,256 (94.4) |
| Multiple sexual partners in the 12 months before the survey, n (%) | 829 (100.0) | 857 (100.0) | 1,686 (100.0) |
| Yes | 109 (15.0) | 117 (15.0) | 226 (15.0) |
| No | 720 (85.0) | 740 (85.0) | 1,460 (85.0) |
| Number of correctly answered HIV knowledge questions, n (%) | 426 (100.0) | 450 (100.0) | 876 (100.0) |
| 0-3 | 200 (47.4) | 218 (47.4) | 418 (47.4) |
| 4 | 210 (49.1) | 220 (50.1) | 430 (49.7) |
| 5 | 16 (3.5) | 12 (2.5) | 28 (3.0) |
| Comprehensive HIV knowledge, n (%) | 426 (100.0) | 450 (100.0) | 876 (100.0) |
| No | 410 (96.5) | 438 (97.5) | 848 (97.0) |
| Yes | 16 (3.5) | 12 (2.5) | 28 (3.0) |
| Discriminatory attitudes toward people living with HIV, n (%) | 426 (100.0) | 447 (100.0) | 873 (100.0) |
| No | 371 (86.7) | 379 (85.6) | 750 (86.1) |
| Yes | 55 (13.3) | 68 (14.4) | 123 (13.9) |
| Any perceived stigma, n (%) | 421 (100.0) | 440 (100.0) | 861 (100.0) |
| No | 65 (14.3) | 66 (16.7) | 131 (15.6) |
| Yes | 356 (85.7) | 374 (83.3) | 730 (84.4) |

^a^Data are number of participants and (weighted percentages).
