# Supplementary material for: Prevalence of and factors associated with late diagnosis of HIV in Malawi, Zambia, and Zimbabwe: Results from population-based nationally representative surveys
Source: PLOS Glob Public Health. 2022 Feb 22;2(2):e0000080. doi: 10.1371/journal.pgph.0000080 (PMC10021857; doi:10.1371/journal.pgph.0000080)
Supplement: S2 Table — (DOCX) [file pgph.0000080.s003.docx]

**S2 Table: Estimated median (interquartile range) time (years) since seroconversion among men and women newly diagnosed with HIV during the survey in Malawi, Zambia, and Zimbabwe (2015–2016)^a^**

|  | **Sex** | | |  |  |
| --- | --- | --- | --- | --- | --- |
|  | Male  N=700 |  | Female  N=1,104 |  | **Total**  N=1,804 |
| Malawi | 2.9 (0.4-3.7) |  | 3.2 (0.4-4.0) |  | 3.0 (0.4-3.8) |
| Zambia | 3.4 (0.4-4.5) |  | 2.8 (0.4-3.9) |  | 3.1 (0.4-4.1) |
| Zimbabwe | 4.1 (1.8-4.7) |  | 3.5 (0.4-4.4) |  | 3.9 (1.1-4.6) |
| Total | 3.7 (0.9-4.3) |  | 3.3 (0.4-4.1) |  | 3.4 (0.4-4.2) |

^a^Time since seroconversion was estimated based on a CD4 depletion model.(15,16)
