# Supplementary material for: Prevalence of and factors associated with late diagnosis of HIV in Malawi, Zambia, and Zimbabwe: Results from population-based nationally representative surveys
Source: PLOS Glob Public Health. 2022 Feb 22;2(2):e0000080. doi: 10.1371/journal.pgph.0000080 (PMC10021857; doi:10.1371/journal.pgph.0000080)
Supplement: S3 Table — (DOCX) [file pgph.0000080.s004.docx]

**S3 Table: Unadjusted analysis of sociodemographic factors associated with late diagnosis of HIV in Malawi, Zambia, and Zimbabwe (2015–2016)**

|  | **OR (95% CI)** | **p-value**^a^ |
| --- | --- | --- |
| Country |  | 0.072 |
| Malawi | 1.00 |  |
| Zambia | 0.93 (0.66-1.30) |  |
| Zimbabwe | 1.26 (0.91-1.75) |  |
| Residence |  | 0.635 |
| Urban | 1.00 |  |
| Rural | 0.94 (0.74-1.20) |  |
| Sex |  | 0.000 |
| Male | 1.00 |  |
| Female | 0.64 (0.51-0.81) |  |
| Age, years |  | 0.000 |
| 15-24 | 1.00 |  |
| 25-34 | 1.68 (1.16-2.42) |  |
| 35-44 | 2.15 (1.47-3.15) |  |
| 45-65 | 2.37 (1.61-3.49) |  |
| Marital status |  | 0.007 |
| Never married | 1.00 |  |
| Married or living together | 1.53 (1.09-2.14) |  |
| Divorced, separated | 1.47 (0.97-2.23) |  |
| Widowed | 2.38 (1.43-3.99) |  |
| Education |  | 0.977 |
| No or primary | 1.00 |  |
| Secondary or higher | 1.00 (0.77-1.29) |  |
| Wealth quintile |  | 0.772 |
| Lowest | 1.00 |  |
| Second | 0.98 (0.66-1.45) |  |
| Middle | 0.90 (0.59-1.37) |  |
| Fourth | 0.88 (0.60-1.30) |  |
| Highest | 1.10 (0.72-1.69) |  |

^a^P-values of categorical variables are for joint test for significance.

Abbreviations: OR, odds ratio; CI, confidence interval.
