# Supplementary material for: Prevalence of and factors associated with late diagnosis of HIV in Malawi, Zambia, and Zimbabwe: Results from population-based nationally representative surveys
Source: PLOS Glob Public Health. 2022 Feb 22;2(2):e0000080. doi: 10.1371/journal.pgph.0000080 (PMC10021857; doi:10.1371/journal.pgph.0000080)
Supplement: S4 Table — (DOCX) [file pgph.0000080.s005.docx]

**S4 Table: Unadjusted analysis of associations between sexual risk behavior, HIV knowledge, and HIV-related stigma and late diagnosis of HIV in Malawi, Zambia, and Zimbabwe (2015–2016)**

|  | **OR (95% CI)** | **p-value^a^** |
| --- | --- | --- |
| Buying or selling sex in the 12 months before the survey |  | 0.678 |
| Yes | 1.13 (0.63-2.03) |  |
| No | 1.00 |  |
| Multiple sexual partners in the 12 months before the survey |  | 0.971 |
| Yes | 1.01 (0.69-1.46) |  |
| No | 1.00 |  |
| Number of correctly answered HIV knowledge questions |  | 0.945 |
| 0-3 | 1.01 (0.64-1.61) |  |
| 4 | 1.07 (0.71-1.60) |  |
| 5 | 1.00 |  |
| Comprehensive HIV knowledge |  | 0.797 |
| No | 1.05 (0.73-1.50) |  |
| Yes | 1.00 |  |
| Any discriminatory attitudes towards people living with HIV |  | 0.716 |
| No | 1.00 |  |
| Yes | 0.91 (0.55-1.50) |  |
| Perceived stigma |  | 0.474 |
| No | 1.00 |  |
| Yes | 1.20 (0.73-1.98) |  |

^a^P-values of categorical variables are for joint test for significance.

Abbreviations: OR, odds ratio; CI, confidence interval.
